# Barriers and Facilitators to the Recruitment and Engagement of Diverse Populations Into Patient and Family Advisory Councils: A Scoping Review

**DOI:** 10.1177/23743735251376068

**Published:** 2025-09-15

**Authors:** Madison P Leia, Kaitlin See, Colleen Cuthbert

**Affiliations:** 1 2129 University of Calgary, Department of Nursing, Calgary, AB, Canada; 2 2129 University of Calgary, Department of Nursing; Department of Oncology, Cumming School of Medicine, Calgary, AB, Canada

**Keywords:** patient and family advisory council, patient engagement, community engagement, diversity, inclusion, equity, shared decision-making

## Abstract

Patient and family advisory councils (PFACs) serve as structured collaborative groups where patients and caregivers partner with healthcare professionals to shape policies, service delivery, and research. Despite guidelines emphasizing the need for diverse representation, PFACs often remain socio-demographically homogenous, excluding vulnerable populations from critical discussions that shape healthcare outcomes. This scoping review examines barriers and facilitators influencing the recruitment and engagement of diverse populations in PFACs. A systematic search identified studies focusing on recruitment and engagement barriers and facilitators targeting under-represented groups. Forty-three studies that met the inclusion criteria were included in the review. Findings reveal that while race/ethnicity, socioeconomic status, and age are commonly considered diversity factors, other key populations such as individuals with disabilities, migrants, and those with lived experiences of homelessness, are often overlooked. Facilitators that can improve reach to these vulnerable populations include culturally tailored outreach, relationship-building with community leaders, and reducing logistical barriers. This review provides actionable recommendations for improving diversity in PFACs, ensuring equitable patient engagement that reflects the full spectrum of healthcare experiences.

## Introduction

At the heart of patient engagement is ensuring “patients’ values, preferences, and needs are heard, understood, and included” by those with the capacity to influence their care.^1^ Thus, the voices that are, or are not, included will inequitably influence the delivery of health services and potentially affect patient outcomes. Patient and family advisory councils (PFACs), one of the most active forms of patient engagement,^2^ are structured collaborative groups where patients and family members partner with health care professionals, administrators, or researchers to provide input and direction to policies, service delivery, and the research process.

Members of PFACs consistently report that diversity is important and often note a desire for greater diversity of membership.^3,4^ Guidelines explicitly state membership should be diverse, specifically that PFACs should reflect the communities they represent.^5–8^ Ensuring diversity in patient engagement through PFACs is especially critical for addressing inequalities in healthcare as the exclusion of diverse members engenders research, policy, and health services initiatives that may not be generalizable to these groups and may further contribute to their marginalization in the healthcare system.^9–10^

Unfortunately, the limited information on PFAC membership indicates that it is often skewed towards socio-demographically privileged members.^11,12^ Furthermore, many PFAC organizers lack knowledge on how to build and sustain collaborative relationships with members of under-represented population groups. Previous research describing best practices in the formation and operation of similar patient engagement groups have either focused on particular settings, such as pediatric hospitals,^13^ or failed to address the inclusion of diverse populations.^14,15^ One recent review documented recruitment strategies to increase diverse representation but did not address long-term engagement.^16^

In addition, no review has described the types and variety of populations included in this literature, limiting our understanding of the full scope and limitations of previous findings. By specifying which populations are represented in this literature, researchers and practitioners can identify more precisely the population groups missing from outreach efforts. This will highlight which particular groups require further research or engagement efforts.

Thus, the research objectives of this scoping review are to (1) identify the populations that have been included in efforts to increase diverse representation in PFACs, and (2) synthesize the existing literature on the barriers preventing and the facilitators supporting recruitment and engagement, with a focus on enhancing diversity within broader communities. This review considers both the representation of specific demographic groups as populations and the broader contexts in which these diverse populations come together and engage as a community.

## Methods

This study followed Levac, Colquon, and O’Brien's updated methodological framework for conducting scoping reviews,^17^ and results are reported per the Preferred Reporting Items for Systematic Reviews and Meta-Analyses extension for Scoping Reviews (PRISMA-ScR) in Table 3 in the Supplementary material.^18^ The search strategy was developed in consultation with a health sciences librarian and iteratively refined to ensure validity and proper range of scope. Subject headings and keywords related to patient, family, caregiver advisory committees, and diversity were combined with keywords related to recruitment, retention, and engagement. The MEDLINE search results are provided in Table 4 in the Supplementary material. Five electronic databases—CINAHL, MEDLINE, PsycINFO, EMBASE, and Scopus—were searched from database inception to July 19, 2023. Gray literature was searched following CADTH guidelines on Google.^19^ Reference lists of included articles were manually searched for additional articles to screen.

English language, full-text, peer-reviewed articles from any country (excluding editorials and protocols) were eligible for inclusion. Studies and gray literature presenting qualitative or quantitative findings from an organization or member perspective on the recruitment and/or engagement of under-represented populations into PFACs were included. Articles using alternative terminology to PFACs such as “boards,” “committees,” “groups,” etcetera were also included, if the entities filled the same function. PFACs that disbanded after a single, specific project were excluded to better understand the challenges and solutions related to retention and engagement. Scoping and systematic reviews were also excluded; however, their reference lists were searched for additional articles to screen. There was no limit on the timeframe for included studies.

Following deduplication per Bramer et al guidelines,^20^ records were uploaded to Covidence systematic review software for screening. Two researchers screened each record independently and in duplicate and repeated the process for full-text articles. Disagreements were resolved through discussion. Data was independently extracted from included studies by 2 researchers, with 1 researcher checking for discrepancies. Study characteristics, population(s) of interest for recruitment or engagement, and barriers and facilitators to recruitment and engagement were extracted using a standardized extraction form.

For the first research objective, data extracted on the population(s) of interest were quantitatively analyzed to produce frequency counts. For the second research objective, barriers and facilitators were qualitatively analyzed by 2 researchers using NVivo 14. After coding line-by-line all extracted text, the researchers grouped codes together to generate descriptive themes. To make findings accessible to healthcare researchers, policymakers, and administrators, a list of concrete recommendations was constructed to accompany themes.

## Results

**Figure 1.**
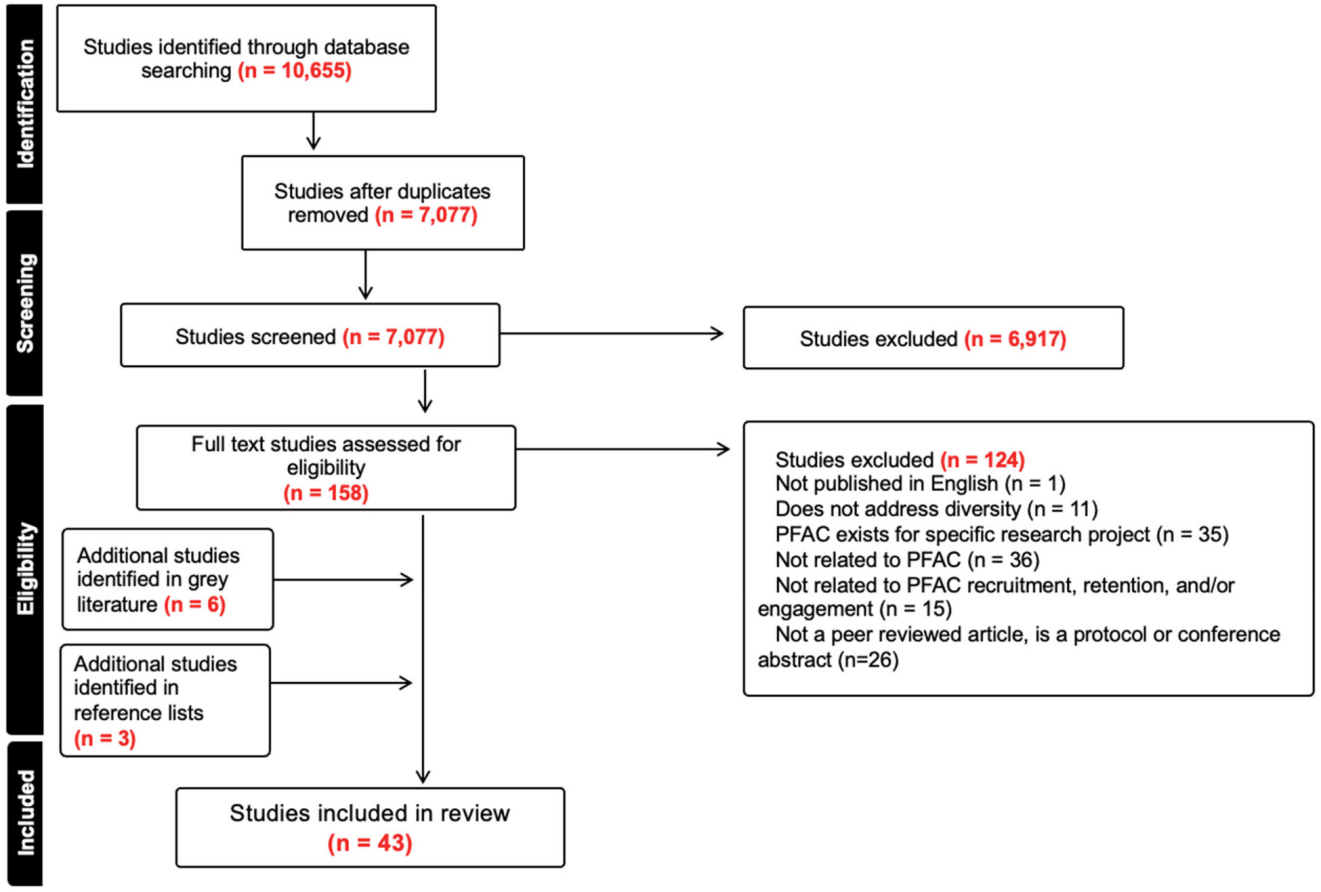
PRISMA flow diagram.

The search yielded a total of 10,655 records, of which 3,578 were identified as duplicates. After title and abstract screening, 6,917 of the 7,077 unique records did not meet eligibility criteria. One hundred and fifty-eight studies were reviewed at the full text stage, of which 124 were excluded. Nine additional studies were identified: 6 through systematic gray literature searching and 3 from reference lists of included articles. In total, 43 studies were included in this review. Figure 1 details the process of study identification using a PRISMA flow chart.

### Study Characteristics

A breakdown of summarized study characteristics is provided in Table 1 in the Supplementary material (citations can be found in Table 2 in the Supplementary material). As shown, almost all included studies were published in Western countries, including the United States (*n* = 31, 72%), Canada (*n* = 5, 11.6%), Australia (*n* = 2, 4.7%), and New Zealand (*n* = 2, 4.7%). Close to half (*n* = 20, 46.5%) were published since 2019, with another 39.5% (*n* = 17) published between 2014 and 2018. PFACs in included studies were formed to advise and provide input primarily to research (*n* = 29) as well as health services (*n* = 12) and policy matters (*n* = 4).

### Populations of Interest

Racial and ethnic populations were the most frequently considered for inclusion, with 79.1% (*n* = 34) of articles referencing either generic categories, such as “ethnic minority populations,”^21^ or specific subpopulations, such as “African Americans.”^22^ Age was the next most mentioned population, appearing in 37% (*n* = 16) of articles, followed by socioeconomic status (32.6%, *n* = 14), gender (25.6%, *n* = 11), geography (23.3%, *n* = 10), and sexual orientation, (20.9%, *n* = 9). As seen in Table 1, less frequently mentioned populations included those defined by disability status, language, religion, migration status, sex, mental illness, and veteran status.

**Table 1.** Populations Considered for PFAC Recruitment and Engagement.

Category	Subcategory	Total
Racial and/or ethnic groups		34
	Black or of African Descent	14
	Latino or Hispanic	9
	Indigenous	8
	Asian	1
	White	1
	Not specified	16
Age		16
	Youth	9
	Advanced age	5
	Not specified	8
Geography		10
	Rural/Remote	6
	Inner city or urban	2
	Not specified	4
Sexual orientation		9
Migration status		4
	Immigrant	3
	Refugee	1
Disability status		6
Mental illness		2
Sex		2
Gender		11
Socioeconomic status		14
	Low income	5
	Education level	4
	Low literacy	1
	Not specified	8
Religion		4
	Islam	1
	Not specified	3
Language		5
	Limited English	2
	Not specified	4
Special populations: veterans		2
Generic terms of diversity		9

Articles which listed generic categories (eg, “racial and/or ethnic groups”) or broad diversity phrases (eg, “vulnerable populations”) in addition to specific subcategories (eg, “Indigenous”) were counted in each respective classification. PFAC, patient and family advisory council.

Most articles (*n* = 24, 55.8%) included more than one specific population of interest, while others (*n* = 9, 20.9%) relied primarily on broad diversity terminology, such as “historically marginalized,”^4,23^ “vulnerable,”^24,25^ or “hard to reach.”^26^ However, among the latter articles, only three relied solely on broad terms without also listing specific categories. One of these three focused simply on “equity, diversity, and inclusion.”^27^ Of the other two, one focused on groups described as “under-represented” and “not connected” to academia, research, or healthcare institutions^28^ and the other on “structurally seldom-heard” groups.^29^

### Recruitment Barriers

Findings indicated that several components of recruitment can alienate or even systematically exclude under-represented individuals. Literacy requirements can prevent typical community members from joining,^24^ while application questions on education or experience were noted as irrelevant as well as “demeaning and exclusionary.”^4^ Another barrier cited was inadequate community knowledge and awareness of the PFAC due to the repeated use of the same individuals or channels in recruitment—a strategy that results in only a subset of the community being reached.^9,30–32^

Mistrust of health institutions manifested as expectations of disrespect,^4,33^ or with some potential members “feeling powerless or that they lack influence.”^9^ In addition, individuals from some communities did not feel comfortable participating in PFACs that focused on a stigmatized condition (eg, mental illness)^33^ or self-identifying as a member of a vulnerable group (eg, sex workers) where doing so could carry legal or social repercussions.^31^ Some cited insufficient time and resources as a barrier,^32^ while others stated that a lack of transportation^9,10,31,34^ or the frequency or distance required to attend meetings deterred them.^34,35^

### Recruitment Facilitators

Recruitment materials explicitly calling for diversity, adapted to reflect the culture and language of specific communities, and with clearly stated accommodations, can help communities recognize themselves as the target audience and evaluate accessibility.^9,10,23,32,36–38^ Building a relationship with the community prior to recruitment, whether through attendance at community events or volunteer work, is helpful for building trust^22,39^ as it dispels the belief that the PFAC is “merely using the community as a resource.”^30^ Volunteering with community organizations also demonstrated sustained commitment beyond the scope of the project.^40^

Seeking referrals or recruiting directly through community organizations facilitates the recruitment of diverse individuals since PFACs can work, “within existing relationships of trust and rapport.”^4,33,41,42^ In one PFAC, the selection of members by the local community organization led to better meeting attendance, group cohesion, and gender parity.^30^ Personal invitations from current members, from healthcare providers associated with the PFAC's parent organization, and from providers serving specific populations benefited similarly from these existing relationships.^37,38,43–45^

Lastly, recruiting in third spaces—including parks, coffee shops, and social media platforms—facilitates participation as it reaches many individuals, including those less engaged with the healthcare system and those who have not previously participated in community organizations, either by choice or due to specific barriers.^32,41,42,46,47^ Additional details on recruitment barriers and facilitators are provided in Table 2.

**Table 2.** Recruitment Barriers and Facilitators.

		Explanation and Recommendations
Barriers	Exclusionary materials and criteria	Recruitment criteria requiring English language fluency or literacy Including questions about education and/or experience on applications Culturally inappropriate recruitment materials Use of jargon in communication
	Historical mistrust and stigma	Potential members expect to be disrespected Certain identities and health concerns may be stigmatized in the community
	No community knowledge of the PFAC	Community members are not aware of the PFAC, what the advisor role is or how to propose new members Stagnant recruitment methods only reaching the same groups
	Geographic distance	Individuals may be unwilling to travel long distances, or have unreliable transportation Efforts to recruit may not reach remote or geographically isolated communities
	Conflicting with primary responsibilities	Individuals may have caregiving responsibilities Meetings may conflict with work schedules
Facilitators	Developing a relationship with community prior to recruitment	Participate in community cultural and social events Volunteer time, services, and support in community settings and organizations, such as assisting with fundraisers or grant applications, providing health education or screenings Use these opportunities to share information on the PFAC with potential members in community spaces, rather than expecting them to enter academic or healthcare institutions
	Understanding the diversity needs of your PFAC	Collect data or use existing patient population and/or community demographics to determine what groups should be represented on the PFAC to be reflective of the community served Use tools such as the Health Equity Impact Assessment^23^ to understand which groups will be impacted by research, policy, or service delivery and should be represented in the PFAC Survey potential members on basic demographic information and use information to assist with selection of group members
	Providing appropriate and relevant information in ads	Describe how the PFAC has addressed possible barriers to participation (eg, caregiving responsibilities, language, transportation) Be explicit about seeking diverse members, and use photos or images that reflect desired or actual community diversity Provide easy to understand descriptions of the role including responsibilities and criteria for membership Translate ads/recruitment materials into language(s) of community Work with community leaders or local artists on how to create materials that speak to diverse cultural perspectives
	Employing healthcare organization driven recruitment methods	Ask clinicians, staff, and other allied health professionals from the hospital or practice (or recruit providers from clinics serving disadvantaged groups) to discuss the PFAC with diverse patients and families, give out personal invitations to join, or identify and recommend potential members to organizers Consider approaching patients and family members who have previously participated in research studies Use practice or organization communication channels (website, social media, internal newsletters, patient surveys, practice mail-outs, practice listservs, posters and brochures in organization's buildings, on TV monitors, and at on-site patient and family support groups) to distribute recruitment ads
	Employing community driven recruitment methods	Recruit from staff or leaders of local community organizations as members, such as tribal committees, faith based organizations, social service agencies like food banks Ask prospective and current members to recommend additional diverse members from their networks Approach individuals at community organizations or peer led support groups (particularly those providing services to specific subpopulations) or ask local organizations to identify and refer typical users of their services Ask local organizations to advertise the opportunity to their members or for insight on how to reach out more effectively to desired subpopulations Disseminate recruitment ads or open meeting notices in local media, particularly those that cater to special/minority populations, on community bulletin boards or newsletters, and in shared public spaces like grocery stores, hair salons, hardware stores, or coffee shops Access digital communities by creating websites or social media accounts for the PFAC and posting recruitment ads in online groups and on social media platforms

PFAC, patient and family advisory council.

### Engagement Barriers

Although virtual meetings can reduce the abovementioned barriers, members in rural or economically disadvantaged areas may still face challenges if they lack reliable internet access.^4,10,47^ Discomfort or limited skills with programs, such as virtual conferencing platforms,^4,10,48^ can interfere with members’ ability to prepare for meetings or provide feedback due to an inability to access or reference materials shared electronically.^47^

Some members may feel unprepared to engage if they lack clarity on the scope of their roles and responsibilities, while others may inadvertently overstep the boundaries of their roles in ways that could be detrimental to the project goals or the broader community.^24^ Communication issues, such as limited English fluency or scientific literacy, also hinder understanding of PFAC materials and may impede meeting efficiency and productivity.^31^

One of the most significant barriers mentioned was the perception that PFAC involvement is tokenistic—a perception that heightened feelings of exclusion and was theorized to contribute to member attrition.^9^ One flagrant example was the invalidation of members’ expertise, especially when it differed from Western forms of evidence. The Indigenous leaders in one study highlighted such behavior, noting that “Maori and Pasifika knowledge and expertise was frequently ignored, debated, contested, or perceived as unworthy or invalid.”^49^ This dynamic was considered challenging to overcome and hindered the respectful exchange of ideas.^48,49^ Furthermore, tokenism reinforced the mistrust that many under-represented communities harbor towards healthcare, government, and research institutions.^33,42^ As several studies indicated, even when individuals from these communities join PFACs, they may hesitate to disclose sensitive personal information due to lingering skepticism about organizers’ intentions.^26,28,50^

### Engagement Facilitators

A consistent byproduct of the engagement facilitators identified was establishing trust between members and organizers. This included frequently scheduled meetings in which members were given the opportunity to ask questions and observe the research process,^28^ a course of action that “helped to demystify the work being conducted”^50^ and engender a sense of mutual respect.^50,51^ Trust was also the most cited output of relationship building efforts,^10,26,36,45^ particularly when organizers shared the personal motivations behind their work.^10,28,52^ Such actions served to break down “real and perceived hierarchies,”^26^ “stereotypes,”^28^ and “dividing lines”^52^ between members and PFAC leaders.

Continually re-orienting members, such as frequently reviewing the PFAC's mission, helped to “sustain focus and momentum,”^46^ while trust was further sustained by transparency surrounding the expectations and processes of the PFAC.^33,48,53^ These effects were similarly recognized when PFACs dispersed authority through shared leadership and evaluation opportunities,^24,27,42,53^ a strategy that promoted power sharing and a sense of equity between members and organizers.^10,25,33,39,42,48,49,54–57^

Another key facilitator was recognizing the validity of non-Western forms of knowledge.^49,50,54^ For example, one PFAC incorporated the Pacific research paradigm “fa’afaletui” into discussions to reframe research questions in a way that encouraged deeper reflection.^43^ Acknowledging local and Indigenous knowledge both empowered members and demonstrated respect.^49,54^

Other supportive actions included maintaining consistent contact with members to increase their comfort when providing input^9,24,33,40,51,53,56,58^ and appointing trained facilitators to help navigate conflict within the group and ensure all members receive equal opportunity to contribute.^25,28,31,47^ PFAC managers who had been coached on how to present complex information to individuals with differing levels of scientific literacy created a safe space for members to comfortably engage,^9,54^ and adopting a collegiate approach helped to further establish a collaborative relationship between members and organizers.^48^ Acknowledging all feedback was a way of letting members know their voices were “heard and welcomed,”^28^ and hosting meetings in familiar, accessible, community-based settings provided a safe physical space for them to meet.^22,33,41^ Lastly, training and orientation activities provided the skills and knowledge necessary for members to participate productively, make informed decisions,^10,28,31,50,57^ and feel confident in the process.^28,47^ Table 3 provides additional details on engagement barriers and facilitators.

**Table 3.** Engagement Barriers and Facilitators.

		Explanation and Recommendations
Barriers	Inadequate training	Lack of instruction or clarity on member roles and responsibilities, as well as role specific training
	Discomfort with or lack of access to technology	Lack of technology or internet access to participate in virtual meetings or to receive electronic materials Those with access may be unfamiliar or uncomfortable using virtual platforms, email, or other software programs
	Tokenism	Inauthentic or shallow engagement with cultural practices Seeking member endorsement with little or no opportunity to provide substantive input on PFAC decisions or direction Expecting a few individuals to represent all diverse perspectives Ignoring or questioning non-Western forms of knowledge including not recording member input
	Mistrust	Suspicion of organizer motives due to historical mistreatment deters open participation
	Difficulty understanding and communicating with staff	Low scientific or health literacy creates extra communication challenges Members who are not proficient in English struggle to understand research materials and productively engage during meetings
Facilitators	Maintaining consistent contact with members	Select a dedicated facilitator to handle PFAC coordination, centralize communication, and act as liaison between members and organizers Adopt an open door policy for communication—including individual meetings if requested—and ensure regular communication using member preferred means Hold consistent and frequent meetings throughout project or member term length
	Recognizing the unique expertise members bring to the PFAC	Be receptive to non-traditional forms of knowledge sharing and consult on culturally inclusive practices to ensure authentic sharing of expertise Track and regularly share success of PFAC and highlight how member input has been utilized Offer letters of thanks and appreciation or celebratory dinners to recognize accomplishments
	Preparing staff to interact with diverse lay collaborators	Assess staff levels of inclusivity and unconscious bias; encourage staff to reflect on their social positioning and how it may influence their engagement with members Provide cultural competency or diversity training and education on relevant history, knowledge systems, and cultural protocols Prepare external presenters for meetings ahead of time; discuss desired level of involvement and outcomes, instruct on how to present information in an accessible manner, set reasonable expectations, encourage a collegiate approach and to acknowledge members feedback
	Preparing members to effectively participate	Provide relevant training to all members, which may include project or practice specific knowledge, research ethics, and information on the research process and methodologies Continually orient members to PFAC purpose, goals, timeline, agendas, their role and responsibilities Set or co-develop clear expectations of behavior (codes of conduct) and be transparent regarding the PFAC role in the organization
	Removing barriers to participation	Reduce direct and indirect costs to participate by offering gift cards or stipends, transportation or parking vouchers, childcare, and/or meals Provide interpreters or hold meetings in members primary language Create materials that consider health literacy, reading level, and avoid jargon Host meetings in safe, trusted settings accessible by public transportation and for members with disabilities Consider flexibility in degree and length of participation, scheduling of, and frequency of meetings Be cautious whether virtual meetings remove barriers or are a barrier to participation
	Focusing on building personal relationships with and between members	Devote time during meetings for personal conversations and socializing, typically at the beginning of your meetings or during meals if provided Create ways for members to stay connected between meetings and share personal updates whether through sharing contact information, group text chats, Facebook pages, or structured outreach programs Use icebreaker and team-building activities to encourage members and staff to share life stories, reasons for participation, and other meaningful information about themselves
	Sharing leadership and decision making responsibilities with members	Encourage members to organize, chair, or facilitate meetings, and contribute to meeting agendas Develop the PFAC vision, priorities or goals as a group Co-create formalized agreements which outline PFAC operating procedures or bylaws, roles and responsibilities, decision making processes and overall authority Provide regular opportunities to evaluate PFAC functioning and processes

PFAC, patient and family advisory council.

## Discussion

We conducted a scoping review to identify which under-represented populations PFACs seek to recruit and engage, and to synthesize the factors that facilitate or impede their efforts. To our knowledge, this is the first review to systematically report on the specific population groups that have been the focus of efforts to increase diversity within PFACs and other advisory councils. Another strength of this review is the inclusion of patient engagement literature across a broad spectrum of health concerns, settings, and engagement purposes.

Our findings identified the groups most frequently referenced or sought out for inclusion in PFACs to improve diversity and engagement. The most frequently cited groups were those defined by race/ethnicity, age, and socioeconomic status. This is indicative of their established associations in the health disparities literature and perhaps a direct response to the predominantly white, highly affluent representation in PFACs currently.^11,12^ However, if the defining characteristics of diversity in patient engagement remain focused on these traditional markers, outreach efforts will continue to miss less visible populations and ultimately stagnate. A commitment to valuing diverse perspectives and achieving full representation of their communities requires PFAC organizers to challenge and expand their preconceptions of diversity.

Gaps in representation that we identified included disability status, migrant status, mental illness, and language spoken. A relatively low number of articles mentioned these categories in their approach to increasing diversity in PFACs. Perhaps unsurprisingly, the most vulnerable members of society—persons with lived experiences of addiction, homelessness, and incarceration – were among the groups not explicitly mentioned by any article. Their exclusion from one of the most effective forms of patient engagement will only contribute to their continued marginalization within the healthcare system and poor health outcomes.^59^ Additional research is urgently needed to identify unique concerns among these populations and the approaches needed to facilitate their inclusion. This will likely include trauma-informed approaches similar to those employed with refugee populations.^33,60^

The results of our study also serve to integrate and expand on insights from community-based participatory research, patient stakeholder engagement, and strategies for recruiting minority participants in clinical studies. They confirm, for the first time, many established principles and recommendations previously unexamined in diverse populations and provide actionable steps for implementation. For instance, while previous reviews have noted that inclusion criteria should reflect research goals or expertise in specific areas,^15^ our study highlights the importance of focusing on specific subpopulations of the community who will be impacted by the research. Our results also expand on the information that must be conveyed in recruitment materials rather than merely identifying the most effective channels for distribution.^61^ Notably, our recommendations to develop relationships with the community are consistent with research into the recruitment of ethnic minority groups to clinical trials, particularly the finding that community perception is one of the strongest factors influencing enrollment intention.^62^

Many of the facilitators identified in this review also align with foundational principles of patient engagement and engagement best practices identified by Harrison et al^14^ Our results expand on their recommendations by highlighting the need to provide specific training and education for organizers and presenters that extends beyond inclusivity and cultural competency to include best practices for effective interactions.

Additionally, while previous work has focused on the need for cost reduction, our findings address barriers such as health literacy challenges, the lack of interpreters, and the need for trusted and accessible meeting spaces. By incorporating these expanded strategies, our study aims to provide a more comprehensive and inclusive framework for sustaining meaningful engagement. Although several of these recommendations are directed at researchers, many are equally relevant for healthcare administrators, PFAC coordinators, and policy makers seeking to improve engagement with diverse communities. Lastly, our results provide unique insights into patient recruitment and engagement, such as the need to respect nontraditional forms of knowledge and knowledge sharing, which have previously been noted to build trust with communities who historically have been negatively impacted by research.^63^ Reflective of the post-COVID world, our results also touch on the multivalent impact of virtual participation for these populations.

## Limitations

Our study has several limitations, including the exclusion of non-English articles. As a result, most included studies occurred in Western countries; therefore, recommendations may be limited to this context. We also excluded articles describing PFACs developed for a single project, aiming instead to evaluate sustained engagement practices. This may have limited insights into short-term models of engagement and unique strategies employed within these contexts. The results pertaining to our first objective underscore that the barriers and facilitators identified may be most applicable to the groups that appeared most frequently in the literature.

Although our search strategy was informed by previous reviews and intended to capture various permutations of PFAC related monikers, it is possible that unconventional terminology applicable to the essence of PFACs was missed. However, given the general consistency between our findings and previous reviews conducted in similar contexts, it is unlikely the inclusion of a few additional studies would have significantly impacted results.

Finally, we note that most articles included data presented from the organizers’ point of view, and no data was included from individuals who declined membership or those who joined PFACs and exited prematurely. This may have positively biased our results and led us to overlook additional barriers experienced by diverse individuals. Future research should investigate if the perceptions and beliefs surrounding participation in PFACs among these individuals follow similar themes to those identified in the literature on barriers to minority groups’ clinical trial enrollment. Further studies might also consider whether recruitment and engagement strategies differ across patients with varying healthcare experiences (eg, positive vs negative encounters; chronic vs acute conditions), as these nuances were not specifically addressed in the included literature.

## Conclusion

Ensuring diversity within patient engagement is an extensive but worthwhile process that requires sustained effort. To aid healthcare professionals, researchers, and other stakeholders in facilitating this process, we have presented a comprehensive list of evidence-based recommendations for PFACs that span pre-engagement planning and initial contact to long-term collaboration and have identified the under-represented groups which deserve more attention in patient engagement literature.

## Supplemental Material

sj-docx-1-jpx-10.1177_23743735251376068 - Supplemental material for Barriers and Facilitators to the Recruitment and Engagement of Diverse Populations Into Patient and Family Advisory Councils: A Scoping Review Supplemental material, sj-docx-1-jpx-10.1177_23743735251376068 for Barriers and Facilitators to the Recruitment and Engagement of Diverse Populations Into Patient and Family Advisory Councils: A Scoping Review by Madison P Leia, Kaitlin See and Colleen Cuthbert in Journal of Patient Experience

## References

[1] Snow ME Tweedie K Pederson A . Heard and valued: the development of a model to meaningfully engage marginalized populations in health services planning. BMC Health Serv Res. 2018 Mar 15;18(1):181. doi:10.1186/s12913-018-2969-1. PMID: 29544486; PMCID: PMC5856315. 29544486 PMC5856315

[2] Domecq JP Prutsky G Elraiyah T , et al. Patient engagement in research: a systematic review. BMC Health Serv Res. 2014;14(89) 10.1186/1472-6963-14-89. PMC3938901 24568690

[3] Bellows M Kovacs Burns K Jackson K Surgeoner B Gallivan J . Meaningful and effective patient engagement: what matters most to stakeholders. Patient Experience Journal. 2015;2(1):18–28. doi:10.35680/2372-0247.1069

[4] Unaka NI Hoang M Hsu J , et al. The intersection of diversity, equity, and inclusion with pediatric patient and family advisory councils. Patient Experience Journal. 2022;9(3):39–54. doi:10.35680/2372-0247.1720

[5] American Hospital Association . Patient and Family Advisory Councils Blueprint: A Start-Up Map and Strategy Guide. January 2022. Retrieved from https://www.aha.org/system/files/media/file/2022/01/alliance-pfac-blueprint-2022.pdf.

[6] Alberta SPOR Support Unit . Patient engagement in health research: A how-to guide for researchers. May 2018. v 8.0. retrieved from https://absporu.ca/wp- content/uploads/2020/05/How-To-Guide-Researcher-Version-8.0-May-2018-1.pdf.

[7] American Medical Association . Forming a Patient and Family Advisory Council. AMA STEPS Forward https://edhub.ama-assn.org/steps-forward/module/2702594.

[8] Agency for Healthcare Research and Quality (AHRQ) Strategy 1: Working with Patients and Families as Advisors. Agency for Healthcare Research and Quality; 2013.

[9] Institute for Patient- and Family-Centered Care . Diverse voices matter: improving diversity in patient and family advisory councils. https://www.ipfcc.org/resources/Diverse-Voices-Matter.pdf. January 2018. Accessed June 6, 2023.

[10] Charlot M Carolan K Gawuga C Freeman E Sprague Martinez L . Patient powered research: an approach to building capacity for a hardly reached patient population to engage in cancer research. Res Involv Engagem. 2021 Oct 26;7(1):74. 34702359 10.1186/s40900-021-00317-7 PMC8547568

[11] Montalbano A Chadwick S Miller D , et al. Demographic characteristics among members of patient family advisory councils at a pediatric health system. J Patient Exp. 2021 Nov 5;5(8):23743735211049680. 10.1177/23743735211049680 PMC8573511 34778548

[12] Warren M Leamon T Hall A , et al. The role of patient advisory councils in health research: lessons from two provincial councils in Canada. J Patient Exp. 2020 Dec;7(6):898–905. 33457517 10.1177/2374373520909598 PMC7786741

[13] Dardess P Dokken DL Unaka NI , et al. Diversity, equity, and inclusion in patient and family advisory councils: advancing best practice in children's hospitals. J Pediatr Health Care. 2024;38(2):184–93. 38429030 10.1016/j.pedhc.2023.11.006

[14] Harrison JD Auerbach AD Anderson W , et al. Patient stakeholder engagement in research: a narrative review to describe foundational principles and best practice activities. Health Expect. 2019;22(3):307–16. 30761699 10.1111/hex.12873 PMC6543160

[15] Newman SD Andrews JO Magwood GS Jenkins C Cox MJ Williamson DC . Community advisory boards in community-based participatory research: a synthesis of best processes. Prev Chronic Dis. 2011;8(3):A70. PMC3103575 21477510

[16] Gilfoyle M Melro C Koskinas E Salsberg J . Recruitment of patients, carers and members of the public to advisory boards, groups and panels in public and patient involved health research: a scoping review. BMJ Open. 2023;13(10):e072918. doi:10.1136/bmjopen-2023-072918 PMC10582988 37832980

[17] Levac D Colquhoun H O'Brien KK . Scoping studies: advancing the methodology. Implement Sci. 2010;5(69). Published 2010 Sep 20. 10.1186/1748-5908-5-69 PMC2954944 20854677

[18] Tricco AC Lillie E Zarin W , et al. PRISMA Extension for scoping reviews (PRISMA-ScR): checklist and explanation. Ann Intern Med. 2018;169(7):467–73. 30178033 10.7326/M18-0850

[19] Grey Matters: A Tool for Searching Health-related Grey Literature . Canadian Agency for Drugs and Technology in Health; 2024. https://greymatters.cda-amc.ca. Accessed July 17, 2023.

[20] Bramer WM Giustini D de Jonge GB Holland L Bekhuis T . De-duplication of database search results for systematic reviews in EndNote [published correction appears in J med libr assoc. 2017 jan;105(1):111. Doi: 10.5195/jmla.2017.128]. J Med Libr Assoc. 2016;104(3):240–3. 27366130 10.3163/1536-5050.104.3.014 PMC4915647

[21] Chauhan A Leefe J Shé EN Harrison R . Optimising co-design with ethnic minority consumers. Int J Equity Health. 2021;20(240):1–6. 34736455 10.1186/s12939-021-01579-z PMC8567634

[22] Dancy BL Wilbur J Talashek M , et al. Community-based research: barriers to recruitment of African Americans. Nurs Outlook. 2004;52(5):234–40. 15499312 10.1016/j.outlook.2004.04.012

[23] Health Quality Ontario . Creating and sustaining Patient and Family Advisory Councils – Recruiting for diversity. https://www.hqontario.ca/Portals/0/documents/pe/recruiting-diversity-en.pdf. 2017. Accessed August 9, 2023.

[24] Kamuya DM Marsh V Kombe FK , et al. Engaging communities to strengthen research ethics in low-income settings: selection and perceptions of members of a network of representatives in coastal Kenya. Dev World Bioeth. 2013;13(1):10–20. 23433404 10.1111/dewb.12014 PMC3654571

[25] Gonzalez-Guarda RM Jones EJ Cohn E , et al. Advancing nursing science through community advisory boards: working effectively across diverse communities. ANS Adv Nurs Sci. 2017;40(3):278–88. 27930402 10.1097/ANS.0000000000000167 PMC5461216

[26] Harrison JD Anderson WG Fagan M , et al. Patient and family advisory councils for research: recruiting and supporting members from diverse and hard-to-reach communities. J Nurs Adm. 2019;49(10):473–9. 31490796 10.1097/NNA.0000000000000790 PMC10985779

[27] Sayani A Maybee A Manthorne J , et al. Equity-mobilizing partnerships in community (EMPaCT): co-designing patient engagement to promote health equity. Health Q. 2022;24(S):86–92. 10.12927/hcq.2022.26768 35467517

[28] Kaiser BL Thomas GR Bowers BJ . A case study of engaging hard-to-reach participants in the research process: community advisors on research design and strategies (CARDS). Res Nurs Health. 2017;40(1):70–9. 27686421 10.1002/nur.21753 PMC5225082

[29] Savas S Etchegary H Stuckless T , et al. Public interest group on cancer research: a successful patient-researcher partnership in Newfoundland and labrador. Res Involv Engagem. 2022;8(46):1–11. 36057599 10.1186/s40900-022-00380-8 PMC9440646

[30] Shubis K Juma O Sharifu R , et al. Challenges of establishing a community advisory board (CAB) in a low-income, low-resource setting: experiences from Bagamoyo, Tanzania. Health Res Policy Syst. 2009;7(16). 10.1186/1478-4505-7-16 PMC2702270 19534798

[31] Lawrence C Stewart K . The challenge of community representation: lessons from six HIV clinical research community advisory boards in Uganda. J Empir Res Hum Res. 2016;11(4):311–21. 10.1177/1556264616665760 27552841

[32] Gaiser MD Santos J Lord T , et al. Institute on Assets and Social Policy. Patient and Family Advisory Councils: Advancing Culturally Effective Patient-Centered Care. https://heller.brandeis.edu/iere/pdfs/jobs/PFAC.pdf. March 2016. Accessed August 9, 2023.

[33] Miller AB Issa OM Hahn E , et al. Developing advisory boards within community-based participatory approaches to improve mental health among refugee communities. Prog Community Health Partnersh. 2021;15(1):107–16. 33775966 10.1353/cpr.2021.0010

[34] Dardess P Dokken DL Unaka NL , et al. Adapting and responding to a pandemic: patient and family advisory councils in children’s hospitals during COVID-19. Patient Exp J. 2022;9(1):62–71. doi:10.35680/2372-0247.1661

[35] Bougrab N Li D Trachtman H , et al. An electronic health record-based strategy to recruit for a patient advisory council for research: implications for inclusion. J Clin Transl Sci. 2020;4(1):69–72. 32257413 10.1017/cts.2019.433 PMC7103472

[36] Berglas S Vautour N Bell D . Creating a patient and community advisory committee at the Canadian agency for drugs and technologies in health. Int J Technol Assess Health Care. 2021;37(19). 10.1017/S0266462320002251 33478596

[37] Hatlie MJ Washington K . American Medical Association. Forming a patient and family advisory council (PFAC). https://edhub.ama-assn.org/steps-forward/module/2702594. August 31, 2016. Accessed July 28, 2023.

[38] National Institute for Children’s Health Quality . Creating a Patient and Family Advisory Council: A Toolkit for Pediatric Practices. https://nichq.org/wp-content/uploads/2024/09/PFAC-Updated.pdf. Accessed August 14, 2023.

[39] D’Alonzo K . Getting started in CBPR – lessons in building community partnerships for new researchers. Nurs Inq. 2010;17(4):288–92. 10.1111/j.1440-1800.2010.00510.x PMC3203531 21059145

[40] Williams EG Smith MJ Boyd B . Perspective: the role of diversity advisory boards in autism research. Autism. 2023;27(3):864–9. 36336998 10.1177/13623613221133633 PMC10073302

[41] Ali SS Mahoui I Hassoun R , et al. The bay area muslim mental health community advisory board: evaluation of a community based participatory approach. Epidemiol Psychiatr Sci. 2023;32(7). 10.1017/S2045796022000786 PMC9971854 36718769

[42] Weinstein ER Herrera CM Serrano LP , et al. Promoting health equity in HIV prevention and treatment research: a practical guide to establishing, implementing, and sustaining community advisory boards. Ther Adv Infect Dis. 2023;10(1):1–14. 10.1177/20499361231151508 PMC9900661 36755989

[43] Lamont R Fishman T Sanders PF , et al. View from the canoe: co-designing research pacific style. Ann Fam Med. 2020;18(2):172–5. 32152023 10.1370/afm.2497 PMC7062480

[44] Portalupi LB Lewis CL Miller CD , et al. Developing a patient and family research advisory panel to include people with significant disease, multimorbidity and advanced age. Fam Pract. 2017;34(3):364–9. 28122848 10.1093/fampra/cmw138 PMC6080532

[45] DeCamp LR Gregory E Polk S , et al. A voice and a vote: the advisory board experiences of spanish-speaking Latina mothers. Hisp Health Care Int. 2015;13(4):217–26. doi:10.1891/1540-4153.13.4.217 26671562 PMC4751862

[46] Adams AK Scott JR Prince R Williamson A . Using community advisory boards to reduce environmental barriers to health in American Indian communities, Wisconsin, 2007-2012. Prev Chronic Dis. 2014;11(160). 10.5888/pcd11.140014 PMC4170726 25232747

[47] Hyde J Wendleton L Fehling K , et al. Strengthening Excellence in Research through Veteran Engagement (SERVE): Toolkit for Veteran Engagement in Research (Version 1). Veterans Health Administration, Health Services Research and Development. https://www.hsrd.research.va.gov/for_researchers/serve/. 2018. Accessed August 9, 2023.

[48] Brown KM Walker L Kaminstein DS . Building an effective and empowered community advisory board for veterans. J Humanist Psychol. 2020;65(5):1088–1109.

[49] Came H McCreanor T Haenga-Collins M Cornes R . Māori and pasifika leaders’ experiences of government health advisory groups in New Zealand. Kōituitui. 2019;14(1):126–35.

[50] Morales CT Muzquiz LI Howlett K , et al. Partnership with the confederated salish and kootenai tribes: establishing an advisory committee for pharmacogenetic research. Prog Community Health Partnersh. 2016;10(2):173–83. 27346763 10.1353/cpr.2016.0035 PMC5015644

[51] Horowitz CR Arniella A James S Bickell NA . Using community-based participatory research to reduce health disparities in east and central harlem. Mt Sinai J Med. 2004;71(6):368–74. 15592655 PMC4301305

[52] Cooper LA Purnell TS Ibe CA , et al. Reaching for health equity and social justice in Baltimore: the evolution of an academic-community partnership and conceptual framework to address hypertension disparities. Ethn Dis. 2016;26(3):369–78. 27440977 10.18865/ed.26.3.369 PMC4948804

[53] Mitchell J Perry T Rorai V , et al. Building and sustaining a community advisory board of African American older adults as the foundation for volunteer research recruitment and retention in health sciences. Ethn Dis. 2020;30(S2):755–64. 33250622 10.18865/ed.30.S2.755 PMC7683030

[54] Bond C Foley W Askew D . It puts a human face on the researched” – a qualitative evaluation of an indigenous health research governance model. Aust N Z J Public Health. 2016;40(S1):S89–95. 10.1111/1753-6405.12422 26260982

[55] Heck JL Jones EJ Parker JG . Establishment of a community advisory board to address postpartum depression among indigenous women. J Obstet Gynecol Neonatal Nurs. 2023;52(4):320–7. 10.1016/j.jogn.2023.04.007 37290490

[56] Hirschey R Getachew B Coleman K , et al. Development of a community advisory board to guide research about cancer disparities in the black and African American community. Nurs Res. 2023;72(2):123–31. 36450360 10.1097/NNR.0000000000000631 PMC9991945

[57] Vaughn LM Jacquez F Zhen-Duan J . Perspectives of community co-researchers about group dynamics and equitable partnership within a community-academic research team. Health Educ Behav. 2018;45(5):682–9. 29618239 10.1177/1090198118769374

[58] Ortega S McAlvain MS Briant KJ , et al. Perspectives of community advisory board members in a community-academic partnership. J Health Care Poor Underserved. 2018;29(4):1529–43. 30449761 10.1353/hpu.2018.0110 PMC6333479

[59] Williamson G . Healthcare access disparities among marginalized communities. GPHMN. 2024;3(1):11–22. Accessed March 4, 2025. https://forthworthjournals.org/journals/index.php/GPHMN/article/view/98.

[60] Roche P Shimmin C Hickes S , et al. Valuing all voices: refining a trauma-informed, intersectional and critical reflexive framework for patient engagement in health research using a qualitative descriptive approach. Res Involv Engagem. 2020;6(42):1–3. doi:10.1186/s40900-020-00224-2. 32699647 PMC7370500

[61] Hussain-Gambles M Leese B Atkin K Brown J Mason S Tovey P . Involving south Asian patients in clinical trials. Health Technol Assess. 2004;8(42):iii–109. doi:10.3310/hta8420 15488164

[62] Rivers D August EM Sehovic I Green L Quinn B P G . A systematic review of the factors influencing African Americans’ participation in cancer clinical trials. Contemp Clin Trials. 2013;35(2):13–32. doi:10.1016/j.cct.2013.03.007 23557729

[63] Christopher S Watts V McCormick AK Young S . Building and maintaining trust in a community-based participatory research partnership. Am J Public Health. 2008;98(8):1398–406. doi:10.2105/AJPH.2007.125757 18556605 PMC2446462

