# Supplementary material for: Barriers and Facilitators to the Recruitment and Engagement of Diverse Populations Into Patient and Family Advisory Councils: A Scoping Review
Source: J Patient Exp. 2025 Sep 15;12:23743735251376068. doi: 10.1177/23743735251376068 (PMC12437180; doi:10.1177/23743735251376068)
Supplement: sj-docx-1-jpx-10.1177_23743735251376068 - Supplemental material for Barriers and Facilitators to the Recruitment and Engagement of Diverse Populations Into Patient and Family Advisory Councils: A Scoping Review [file sj-docx-1-jpx-10.1177_23743735251376068.docx]

**Supplementary Data File**

**Supplementary Table 1**. Summary Characteristics of Included Studies

| Characteristics | | | No. of Studies | Studies |
| --- | --- | --- | --- | --- |
| Country | | |  |  |
|  | United States | | 31 | [1, 2, 5, 6, 8, 10-17, 19-24, 28-34, 39-43] |
|  | Canada | | 5 | [3, 18, 35-37] |
|  | Australia | | 2 | [4, 9] |
|  | New Zealand | | 2 | [7, 26] |
|  | Kenya | | 1 | [25] |
|  | Tanzania | | 1 | [38] |
|  | Uganda | | 1 | [27] |
| Year Published | | |  |  |
|  | 2004-2008 | | 2 | [12, 21] |
|  | 2009-2013 | | 4 | [11, 25, 31, 38] |
|  | 2014-2018 | | 17 | [1, 4, 10, 14, 15, 17, 18, 22-24, 27, 30, 32-34, 40, 41] |
|  | 2019-2023 | | 20 | [2, 3, 5-9, 13, 16, 19, 20, 26, 28, 29, 35-37, 39, 42, 43] |
| Publication Type | | |  |  |
|  | Empirical study | | 37 | [1-16, 19-21, 24-30, 32-40, 42, 43] |
|  | Grey literature | | 6 | [17, 18, 22, 23, 31, 41] |
| Health Concern | | |  |  |
|  | Specific health concern | |  |  |
|  | | Cancer | 6 | [8, 9, 20, 30, 32, 36] |
|  | | Mental Health | 3 | [2, 19, 28] |
|  | | HIV | 2 | [27, 42] |
|  | | Obesity | 1 | [1] |
|  | | Autism | 1 | [43] |
|  | | Hypertension | 1 | [10] |
|  | | Diabetes | 1 | [32] |
|  | Clinic/hospital specific | |  |  |
|  | | Pediatrics | 6 | [13, 14, 31, 34, 35, 39] |
|  | | General | 3 | [16, 23, 24] |
|  | General health | |  |  |
|  | | Health | 9 | [4, 6, 12, 15, 21, 26, 29, 40, 41] |
|  | | Health Research | 3 | [5, 8, 38] |
|  | | Health Policy | 2 | [3, 7] |
|  | Not specific | | 7 | [11, 17, 18, 22, 25, 33, 37] |
| Study Area | | |  |  |
|  | Research | | 29 | [2, 4-6, 8, 10-12, 15, 16, 19-21, 24-30, 32, 33, 36, 39-43] |
|  | Health services | | 12 | [3, 9, 13, 14, 17, 18, 22, 23, 31, 34, 35, 38] |
|  | Policy | | 4 | [1, 3, 7, 37] |
| PFAC Composition | | |  |  |
|  | Community members | | 25 | [1, 2, 4, 7, 10-12, 15, 19, 21, 24-26, 28-30, 32, 38-40] |
|  | Patients | | 16 | [5, 8, 9, 13, 16-18, 20, 22, 23, 27, 33, 34, 36, 37, 42] |
|  | Family/caregivers | | 15 | [3, 6, 8, 13, 14, 16-18, 22, 23, 31, 34-36, 41] |
|  | Healthcare and/or research staff | | 5 | [16, 34, 35, 41, 43] |

HIV: Human Immunodeficiency Virus, PFAC: Patient and Family Advisory Council

**Supplementary Table 2.** Citations for Included Studies

| Citations for Included Studies |
| --- |
| 1. Adams AK, Scott JR, Prince R, Williamson A. Using community advisory boards to reduce environmental barriers to health in American Indian communities, Wisconsin, 2007-2012. *Prev Chronic Dis*. 2014;11:E160. 2. Ali SS, Mahoui I, Hassoun R, et al. The Bay Area Muslim mental health community advisory board: evaluation of a community based participatory approach. *Epidemiol Psychiatr Sci.* 2023;32:e7 3. Berglas S, Vautour N, Bell D. Creating a patient and community advisory committee at the Canadian Agency for Drugs and Technologies in Health. *Int J Technol Assess Health Care*. 2021;37:e19. 4. Bond C, Foley W, Askew D. “It puts a human face on the researched” – a qualitative evaluation of an Indigenous health research governance model. *Aust N Z J Public Health.* 2016;40(S1):S89-95. 5. Bougrab N, Li D, Trachtman H, et al. An electronic health record-based strategy to recruit for a patient advisory council for research: implications for inclusion. *J Clin Transl Sci.* 2020;4(1):69-72. 6. Brown KM, Walker L, Kaminstein DS. Building an effective and empowered community advisory board for veterans. *J Humanist Psychol.* 2020;0(0):1-22. 7. Came H, McCreanor T, Haenga-Collins M, Cornes R. Māori and Pasifika leaders’ experiences of government health advisory groups in New Zealand. *Kōituitui.* 2019;14(1):126-135. 8. Charlot M, Carolan K, Gawuga C, et al. Patient powered research: an approach to building capacity for a hardly reached patient population to engage in cancer research. *Res Involv Engagem.* 2021;7(74). 9. Chauhan A, Leefe J, Shé EN, Harrison R. Optimising co-design with ethnic minority consumers. *Int J Equity Health.* 2021;20(240). 10. Cooper LA, Purnell TS, Ibe CA, et al. Reaching for health equity and social justice in Baltimore: the evolution of an academic-community partnership and conceptual framework to address hypertension disparities. *Ethn Dis*. 2016;26(3):369-378. 11. D’Alonzo K. Getting started in CBPR – lessons in building community partnerships for new researchers. *Nurs Inq.* 2010;17(4):292-299. 12. Dancy BL, Wilbur J, Talashek M, et al. Community-based research: barriers to recruitment of African Americans. *Nurs Outlook.* 2004;52(5):234-240. 13. Dardess P, Dokken DL, Unaka NL, et al. Adapting and responding to a pandemic: patient and family advisory councils in children’s hospitals during COVID-19. *Patient Exp J.* 2022;9(1): 62-71. doi: 10.35680/2372-0247.1661 14. DeCamp LR, Gregory E, Polk S, et al. A voice and a vote: the advisory board experiences of Spanish-speaking Latina mothers. *Hisp Health Care Int.* 2015;13(4):217-226. doi: 10.1891/1540-4153.13.4.217 15. Gonzalez-Guarda RM, Jones EJ, Cohn E, et al. Advancing nursing science through community advisory boards: working effectively across diverse communities. ANS *Adv Nurs Sci.* 2017;40(3):278-288. 16. Harrison JD, Anderson WG, Fagan M, et al. Patient and family advisory councils for research: recruiting and supporting members from diverse and hard-to-reach communities. *J Nurs Adm.* 2019;49(10): 473-479. 17. Hatlie MJ, Washington K., American Medical Association. Forming a patient and family advisory council (PFAC). <https://edhub.ama-assn.org/steps-forward/module/2702594>. August 31, 2016. 18. Health Quality Ontario. Creating and sustaining Patient and Family Advisory Councils – Recruiting for diversity. <https://www.hqontario.ca/Portals/0/documents/pe/recruiting-diversity-en.pdf>. 2017. 19. Heck JL, Jones EJ, Parker JG. Establishment of a community advisory board to address postpartum depression among Indigenous women. *J Obstet Gynecol Neonatal Nurs.* 2023;52(4):320-327. 20. Hirschey R, Getachew B, Coleman Jr K, et al. Development of a community advisory board to guide research about cancer disparities in the Black and African American community. *Nurs Res.* 2023;72(2):123-131. 21. Horowitz CR, Arniella A, James S, Bickell NA. Using community-based participatory research to reduce health disparities in East and Central Harlem. *Mt Sinai J Med*. 2004;71(6):368-374. 22. Institute for Patient- and Family-Centered Care. Diverse voices matter: improving diversity in patient and family advisory councils. <https://www.ipfcc.org/resources/Diverse-Voices-Matter.pdf>. January 2018. 23. Gaiser MD, Santos J, Lord T, et al. Institute on Assets and Social Policy. Patient and Family Advisory Councils: Advancing Culturally Effective Patient-Centered Care. <https://heller.brandeis.edu/iere/pdfs/jobs/PFAC.pdf>. March 2016. 24. Kaiser BL, Thomas GR, Bowers BJ. A case study of engaging hard-to-reach participants in the research process: community advisors on research design and strategies (CARDS). *Res Nurs Health.* 2017;40(1):70-79. 25. Kamuya DM, Marsh V, Kombe FK, et al. Engaging communities to strengthen research ethics in low-income settings: selection and perceptions of members of a network of representatives in coastal Kenya. *Dev World Bioeth.* 2013;13(1):10-20. 26. Lamont R, Fishman T, Sanders PF, et al. View from the canoe: co-designing research Pacific style. *Ann Fam Med.* 2020;18(2):172-175. 27. Lawrence C, Stewart K. The challenge of community representation: lessons from six HIV clinical research community advisory boards in Uganda. *J Empir Res Hum Res*. 2016;11(4):311-321. 28. Miller AB, Issa OM, Hahn E, et al. Developing advisory boards within community-based participatory approaches to improve mental health among refugee communities. *Prog Community Health Partnersh.* 2021;15(1):107-116. 29. Mitchell J, Perry T, Rorai V, et al. Building and sustaining a community advisory board of African American older adults as the foundation for volunteer research recruitment and retention in health sciences. *Ethn Dis.* 2020;30(S2):755-764. 30. Morales CT, Muzquiz LI, Howlett K, et al. Partnership with the Confederated Salish and Kootenai Tribes: establishing an advisory committee for pharmacogenetic research. *Prog Community Health Partnersh.* 2016;10(2):173-183. 31. National Institute for Children’s Health Quality. Creating a Patient and Family Advisory Council: A Toolkit for Pediatric Practices. <https://nichq.org/wp-content/uploads/2024/09/PFAC-Updated.pdf>. 32. Ortega S, McAlvain MS, Briant KJ, et al. Perspectives of community advisory board members in a community-academic partnership. *J Health Care Poor Underserved.* 2018;29(4):1529-1543. 33. Portalupi LB, Lewis CL, Miller CD, et al. Developing a patient and family research advisory panel to include people with significant disease, multimorbidity and advanced age. *Fam Pract.* 2017;34(3):364-369. 34. Rich C, Goncalves A, Guardiani M, et al. Teen advisory committee: lessons learned by adolescents, facilitators, and hospital staff. *Pediatr Nurs.* 2014;40(6):289-296. 35. Richard J, Azar R, Doucet S, et al. Pediatric patient and family advisory councils: a guide to their development and ongoing implementation. *J Patient Exp.* 2020;7(6): 1476-1481. 36. Savas S, Etchegary H, Stuckless T, et al. Public interest group on cancer research: a successful patient-researcher partnership in Newfoundland and Labrador. *Res Involv Engagem.* 2022;8(46). 37. Sayani A, Maybee A, Manthorne J, et al. Equity-mobilizing partnerships in community (EMPaCT): co-designing patient engagement to promote health equity. *Health Q.* 2022;24(S):86-92. 38. Shubis K, Juma O, Sharifu R, et al. Challenges of establishing a community advisory board (CAB) in a low-income, low-resource setting: experiences from Bagamoyo, Tanzania. *Health Res Policy Syst*. 2009;7:16. 39. Unaka NI, Hoang M, Hsu J, et al. The intersection of diversity, equity, and inclusion with pediatric patient and family advisory councils. *Patient Exp J*. 2022;9(3):39-54. 40. Vaughn LM, Jacquez F, Zhen-Duan J. Perspectives of community co-researchers about group dynamics and equitable partnership within a community-academic research team. *Health Educ Behav.* 2018;45(5): 682-689. 41. Hyde J, Wendleton L, Fehling K, et al. Strengthening Excellence in Research through Veteran Engagement (SERVE): Toolkit for Veteran Engagement in Research (Version 1). Veterans Health Administration, Health Services Research and Development. <https://www.hsrd.research.va.gov/for_researchers/serve/>. 2018. 42. Weinstein ER, Herrera CM, Serrano LP, et al. Promoting health equity in HIV prevention and treatment research: a practical guide to establishing, implementing, and sustaining community advisory boards. *Ther Adv Infect Dis*. 2023;10:1-14. 43. Williams EG, Smith MJ, Boyd B. Perspective: the role of diversity advisory boards in autism research. *Autism*. 2023;27(3):864-869. |

**Supplementary Table 3**. Preferred Reporting Items for Systematic Reviews and Meta-Analyses – Scoping Review (PRISMA-ScR) Checklist

| **SECTION** | **ITEM** | **PRISMA-ScR CHECKLIST ITEM** | **REPORTED ON PAGE #** |
| --- | --- | --- | --- |
| **TITLE** | | | |
| Title | 1 | Identify the report as a scoping review. | Page 1 |
| **ABSTRACT** | | | |
| Structured summary | 2 | Provide a structured summary that includes (as applicable): background, objectives, eligibility criteria, sources of evidence, charting methods, results, and conclusions that relate to the review questions and objectives. | Page 1 |
| **INTRODUCTION** | | | |
| Rationale | 3 | Describe the rationale for the review in the context of what is already known. Explain why the review questions/objectives lend themselves to a scoping review approach. | Pages 2-3 |
| Objectives | 4 | Provide an explicit statement of the questions and objectives being addressed with reference to their key elements (e.g., population or participants, concepts, and context) or other relevant key elements used to conceptualize the review questions and/or objectives. | Page 3 |
| **METHODS** | | | |
| Protocol and registration | 5 | Indicate whether a review protocol exists; state if and where it can be accessed (e.g., a Web address); and if available, provide registration information, including the registration number. | Not applicable |
| Eligibility criteria | 6 | Specify characteristics of the sources of evidence used as eligibility criteria (e.g., years considered, language, and publication status), and provide a rationale. | Page 4 |
| Information sources | 7 | Describe all information sources in the search (e.g., databases with dates of coverage and contact with authors to identify additional sources), as well as the date the most recent search was executed. | Page 4 |
| Search | 8 | Present the full electronic search strategy for at least 1 database, including any limits used, such that it could be repeated. | Supplementary File 1 |
| Selection of sources of evidence | 9 | State the process for selecting sources of evidence (i.e., screening and eligibility) included in the scoping review. | Page 5 |
| Data charting process | 10 | Describe the methods of charting data from the included sources of evidence (e.g., calibrated forms or forms that have been tested by the team before their use, and whether data charting was done independently or in duplicate) and any processes for obtaining and confirming data from investigators. | Page 5 |
| Data items | 11 | List and define all variables for which data were sought and any assumptions and simplifications made. | Page 5 |
| Critical appraisal of individual sources of evidence§ | 12 | If done, provide a rationale for conducting a critical appraisal of included sources of evidence; describe the methods used and how this information was used in any data synthesis (if appropriate). | Not applicable |
| Synthesis of results | 13 | Describe the methods of handling and summarizing the data that were charted. | Pages 5 |
| **RESULTS** | | | |
| Selection of sources of evidence | 14 | Give numbers of sources of evidence screened, assessed for eligibility, and included in the review, with reasons for exclusions at each stage, ideally using a flow diagram. | Page 5, Figure 1 |
| Characteristics of sources of evidence | 15 | For each source of evidence, present characteristics for which data were charted and provide the citations. | Page 6, Supplementary File 1 |
| Critical appraisal within sources of evidence | 16 | If done, present data on critical appraisal of included sources of evidence (see item 12). | Not applicable |
| Results of individual sources of evidence | 17 | For each included source of evidence, present the relevant data that were charted that relate to the review questions and objectives. | Pages 6-11, Table 1, Table 2, Table 3 |
| Synthesis of results | 18 | Summarize and/or present the charting results as they relate to the review questions and objectives. | Pages 6-11, Table 1, Table 2, Table 3 |
| **DISCUSSION** | | | |
| Summary of evidence | 19 | Summarize the main results (including an overview of concepts, themes, and types of evidence available), link to the review questions and objectives, and consider the relevance to key groups. | Pages 11-14 |
| Limitations | 20 | Discuss the limitations of the scoping review process. | Pages 14-15 |
| Conclusions | 21 | Provide a general interpretation of the results with respect to the review questions and objectives, as well as potential implications and/or next steps. | Page 15 |
| **FUNDING** | | | |
| Funding | 22 | Describe sources of funding for the included sources of evidence, as well as sources of funding for the scoping review. Describe the role of the funders of the scoping review. | Not applicable |

*Adapted From:* Tricco AC, Lillie E, Zarin W, O'Brien KK, Colquhoun H, Levac D, et al. PRISMA Extension for Scoping Reviews (PRISMA-ScR): Checklist and Explanation. Ann Intern Med. 2018;169:467–473. [doi: 10.7326/M18-0850](http://annals.org/aim/fullarticle/2700389/prisma-extension-scoping-reviews-prisma-scr-checklist-explanation)

**Supplementary Table 4**. MEDLINE Search Strategy

| MEDLINE Search Strategy |
| --- |
| 1 exp advisory committees/  2 exp community-based participatory research/  3 exp patient participation/  4 exp patient-centered care/  5 ((patient and family) adj advisor*) or (caregiver* adj2 (advisor* or board* or panel* or council* or group* or committee*)) or (family adj2 (advisor* or board* or panel* or council* or group* or committee*)) or (patient* adj2 (advisor* or board* or panel* or council* or committee*)) or "patient partner*".tw.  6 or/1-5  7 exp cultural diversity/  8 exp diversity, equity, inclusion/  9 exp vulnerable populations/  10 exp minority groups/  11 exp gender equity/  12 exp "sexual and gender minorities"/  13 exp health equity/  14 exp social marginalization/  15 exp intersectional framework/  16 exp social determinants of health/  17 exp health inequities/  18 exp minority health/  19 exp "ethnic and racial minorities"/  20 exp "emigrants and immigrants"/  21 exp indigenous peoples/  22 exp indians, north american/  23 exp "american indian or alaska native"/  24 exp inuit/  25 exp social class/  26 "underrepresented" or "under-represented" or diverse or diversity or marginaliz* or vulnerable or equit* or "low income" or lgbt* or edi or dei.tw  27 or/7-26  28 recruit* or engag* or meaningful* or retain*.tw  29 6 and 27 and 28 |
